# Chemical composition and functional properties of roe concentrates from skipjack tuna (*Katsuwonus pelamis*) by cook‐dried process

**DOI:** 10.1002/fsn3.676

**Published:** 2018-05-21

**Authors:** In Seong Yoon, Gyoon‐Woo Lee, Sang In Kang, Sun Young Park, Jung Suck Lee, Jin‐Soo Kim, Min Soo Heu

**Affiliations:** ^1^ Department of Food and Nutrition/Institute of Marine Industry Gyeongsang National University Jinju Korea; ^2^ Department of Seafood and Aquaculture Science/Institute of Marine Industry Gyeongsang National University Tongyeong Korea; ^3^ Research Center for Industrial Development of Seafood Gyeongsang National University Tongyeong Korea

**Keywords:** cook‐dried process, fish roe, food functionality, roe concentrates, skipjack tuna

## Abstract

The objective of this study was to investigate physicochemical properties of protein concentrate from skipjack tuna roe by a cook‐dried (boiled or steamed‐dried) process, and to evaluate their food functional properties. The yields of boil‐dried concentrate (BDC) and steam‐dried concentrate (SDC) prepared from skipjack tuna roe were 22.4 for BDC and 24.4% for SDC. Their protein yields were 16.8 and 18.4%, respectively. In terms of major minerals of the BDC and SDC, sulfur (853.2 and 816.6 mg/100 g) exhibited the highest levels followed by potassium, sodium and phosphorus. The prominent amino acids of roe protein concentrates (RPCs) were Glu, Asp, Leu and Val. The BDC and SDC showed a higher buffer capacity than egg white (EW) at the pH‐shift range. The pH‐shift treatment significantly improved the water holding capacities of RPCs, except pH 6. But they had a low solubility across the pH‐shift range. The foaming capacities (104%–119%) of BDC and SDC were significantly lower than those of EW (*p *<* *.05), and their foam stabilities were not observed. Emulsifying activity index (m^2^/g protein) of RPCs and EW was 2.3 for BDC, 11.1 for SDC and 18.0 for EW. RPCs in the food and seafood processing industries will be available as egg white alternative protein sources and will be available as ingredients of surimi‐based products in particular.

## 1 INTRODUCTION

The skipjack tuna (*Katsuwonus pelamis*) catch is the most abundant of the major commercial tuna species produced in more than 238,732 metric tons in Korean overseas fisheries, and is used widely in raw fish dishes, such as sushi and sliced raw fish fillets (sashimi) in Korea and Japan (Lee et al., 2016). As a canned product, it makes up a total amount of 55,135 metric tons, which accounted for 66% of total canned products in Korea (MOF, 2016).

More than 60% of the seafood processing byproducts are fish waste, including the head, skin, frames, viscera, fat, fins, and roe. In the past, these byproducts were regarded as low values and were used in the production of ensilage or fertilizer, or were thrown away (FAO, 2016). The majority of fishery byproducts are currently utilized in the production of pet food, fish feed, fish oil, fish meal, and fertilizers (Narsing Rao, Balaswamy, Satyanarayana, & Prabhakara Rao, 2012). Among the seafood processing byproducts, fish roes are highly nutritious substances rich in essential amino acids and fatty acids and minerals (Lee et al., 2016; Narsing Rao et al., 2012). Fish roe contains 75% ovoglobulin, 13% collagen and 11% albumins (Sikorski, 1994), was reported to be a good source of nutritional and functional food ingredients. Furthermore, tuna roes are well known as nutritional sources for human consumption, especially polyunsaturated fatty acids (Heu et al., 2006; Intarasirisawat, Benjakul, & Visessanguan, 2011), and functional proteins such as vitellogenin and vitellogenin derivatives (Park et al., 2016). These proteins are found mainly in egg yolk and naturally exist in the granule form of lipovitellin–phosvitin complex with low solubility. The recovery of these valuable components from tuna roe (Heu et al., 2006; Intarasirisawat et al., 2011; Lee et al., 2016; Park et al., 2016) can increase its value‐added and reduce treatment costs or waste disposal. The literature on the physicochemical and functional properties of fish protein utilization from seafood byproducts is available, but data on the characteristics of the roe protein concentrates (RPC) are limited (Lee et al., 2016; Narsing Rao et al., 2012). Thus, intensive research is highly needed to maximize the use of fish roes.

The cooking (boiling and steaming) process of fish and its products improves its digestibility and palatability, and provides safe consumption by killing harmful bacteria and parasites (Lee et al., 2016). The drying process of fish is important because it inactivates enzymes to preserves fish and removes the moisture required for growth of bacteria and fungi (Duan, Jiang, Wang, Yu, & Wang, 2011). The cooking and drying process depend on various processing conditions in the food manufacturing process, thus leading to conformational changes in the protein (Mariod, Fathy, & Ismail, 2010). Such changes may be beneficial or detrimental in terms of the functional or nutritional properties of the processed food system. Thus, processing means are needed to convert the underutilized skipjack tuna roe into more marketable and consumer‐acceptable forms of protein concentrate. Little information is known about the tuna roe protein concentrate by the cook‐dried process (Lee et al., 2016). Protein concentrates can be widely used as ingredients in the food industry due to their high protein levels and nutritional quality, functional properties and low content of anti‐nutritional factors (Narsing Rao, 2014). The use of fish proteins in powder form does not require special storage conditions and is also easy to use as a food ingredient, offering several advantages (Lee et al., 2016; Sathivel, Yin, Bechtel, & King, 2009). The purpose of this study was to investigate the physicochemical properties in terms of proximate composition, amino acids and mineral contents of protein concentrate prepared from skipjack tuna roe by the cook‐dried (boiled or steamed‐dried) process, and to evaluate their food functional properties.

## 2 MATERIALS AND METHODS

### 2.1 Raw sample

Skipjack tuna *Katsuwonus pelamis* roe was purchased from Dongwon F&B Co. Ltd. (Changwon, Korea) and used as experimental material. The roes were sealed in polyethylene bags and stored at −70°C. Frozen roes were partially thawed for 24 hr at 4°C, cut into small pieces about 1.5–3.0 cm thick and ground with a food pulverizer (SFM‐555SP, Shinil Industrial Co. Ltd. Seoul, Korea). The ground roes were stored at −20°C until used.

### 2.2 Chemicals

Bovine serum albumin (BSA), glycerol, β‐mercaptoethanol, sodium hydroxide, and sodium L‐tartrate were purchased from Sigma‐Aldrich Co., LLC. (St. Louis, MO). Coomassie Brilliant Blue R‐250 was purchased from Bio‐Rad Laboratories (Hercules, CA). Copper (II) sulfate pentahydrate, 1 N‐hydrochloric acid and 1 N‐sodium hydroxide were purchased from Yakuri Pure Chemicals Co. Ltd. (Kyoto, Japan). Folin‐Ciocalteu's reagent were purchased from Junsei Chemical Co., Ltd. (Tokyo, Japan). Sodium dodecyl sulfate (SDS) and glycine were purchased from Bio Basic Inc., (Ontario, Canada). Soy bean oil was purchased from Ottogi Co. Ltd. (Seoul, Korea). Trichloroacetic acid was purchased from Kanto Chemical Co. Inc. (Tokyo, Japan). Other reagents used in the experiments were analytical grade.

### 2.3 Preparation of roe protein concentrates

**Figure 1:**
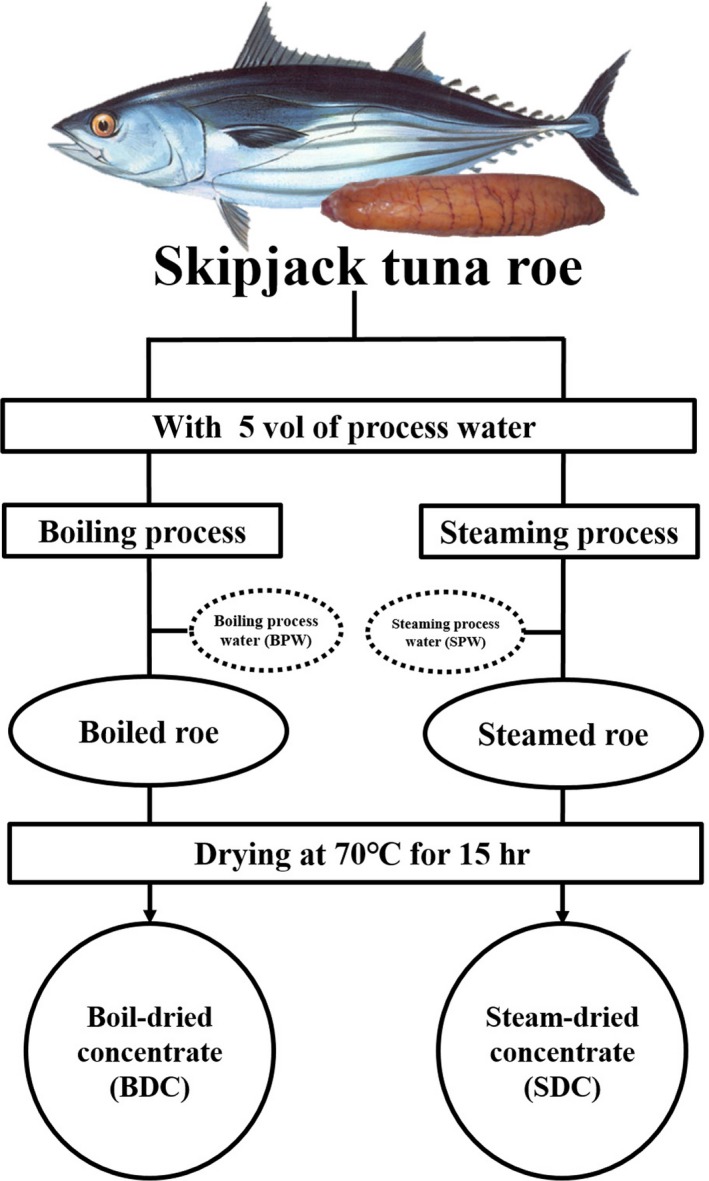
Flowchart for preparation of skipjack tuna roe concentrates by cook‐dried process

RPCs from skipjack tuna roe were prepared by slightly modifying the method of Lee et al. (2016), and its processing is shown in Figure 1. Briefly, 300 g of ground roe were placed in a pouch‐type tea bag (polyethylene polyprophylene, 16 × 14.5 cm) for the cooking and drying process. To prepare the boil‐dried concentrate (BDC), the sample was immerged in 5 volume of deionized distilled water (DDW) and boiled for 20 min after the sample core temperature reached 80°C. For the case of the steam‐dried concentrate (SDC), the sample was steamed for 20 min after the core temperature of the sample reached 80°C. The cooked samples were dried at 70 ± 1°C for 15 hr using an incubator (VS‐1203P3V, Vision Scientific, Co. Ltd. Daejeon, Korea). The boil or steam‐dried samples were pulverized into powders, using a food pulverizer and passed through a 180 mesh sieve. The pulverized powders are referred to as BDC and SDC respectively.

### 2.4 Proximate compositions

The proximate composition including moisture (950.46), crude protein (928.08), crude fat (960.39) and ash content (920.153) were analyzed according to the AOAC method (AOAC, 2000).

### 2.5 Protein concentration

The protein concentration of the samples (1% w/v, dispersion) was determined by the method of Lowry, Rosebrough, Farr, and Randall (1951), using bovine serum albumin as a standard.

### 2.6 Total amino acids

A quantity of 20 mg of samples was hydrolyzed with 2 ml of 6 N HCl in a heating block (HF21, Yamoto Science Co. Tokyo, Japan) at 110°C for 24 hr and filtered, using a vacuum filter (ASPIRATOR A‐3S, EYELA, Tokyo, Japan). The hydrolyzed filtrate was finally diluted with 25 ml of sodium citrate sample buffer and each amino acid was quantified using the amino acid analyzer (model 6300 Biochrom 30, Biochrom Ltd. Cambridge UK) employing sodium citrate buffers (pH 2.2) as stepwise gradients. Amino acid analysis results are expressed as mg of amino acid content per 100 g of protein.

### 2.7 Minerals

Mineral content analysis of the samples was performed, using the inductively coupled plasma optical emission spectrophotometry (OPTIMA 4300 DV, Perkin Elmer, Shelton, CT, USA). A quantity of 100 mg of samples was mixed with 10 ml of 70% (v/v) nitric acid and dissolved and heated on a hot plate until digestion was complete. A total of 5 ml of 2% nitric acid was added to the digested samples, filtered (Advantec No. 2, Toyo Roshi Kaisha, Ltd. Tokyo, Japan), and filtrates were adjusted to 100 ml with 2% nitric acid using a volumetric flask. Mineral concentrations were expressed in mg/100 g samples.

### 2.8 Sodium dodecyl sulfate polyacrylamide gel electrophoresis

The molecular weight patterns of protein were observed with SDS–PAGE, according to the method of Laemmli (1970). Briefly, 20 mg of samples were solubilized in 5 ml of 5% SDS solution. The solubilized samples were mixed with SDS‐PAGE sample treatment buffer (pH 6.8) at a 4:1 (v/v) ratio and boiled at 100°C for 3 min Samples (20 μg protein) were injected into a 10% Mini‐PROTEAN^®^ TGX^™^ Precast gel (Bio‐Rad Lab., Inc.) and electrophoresed at a constant current of 10 mA per gel, using a Mini‐PROTEAN^®^ Tetra cell (Bio‐Rad Lab. Inc.). The molecular weights of the protein bands were estimated using Precision Plus Protein^™^ standards (10–250 K, Bio‐Rad Lab. Inc.).

### 2.9 Buffer capacity

Buffer capacity was measured according to the method of Park et al. (2016). Briefly, 300 mg of samples was dispersed in 30 ml of deionized distilled water and samples in the pH 2–12 range were prepared, adjusting the pH by 1 unit, using 0.5 M NaOH or HCl. The amount of acid and alkaline added for corresponding pH adjustment was recorded and the buffer capacity of samples at each pH was expressed as the mean value of mmol/L of HCl or NaOH per gram of sample required causing a change in pH of 1 unit.

### 2.10 Water holding capacity

The Water holding capacity (WHC) of the samples was measured according to the method of Park et al. (2016). A quantity of 300 mg of sample was placed in a 50 ml centrifuge tube and 30 ml DDW was added. The mixture was thoroughly agitated for 10 min at room temperature and centrifuged at 12,000 g for 20 min at 4°C. The WHC was determined from the difference in weights and expressed as gram of water absorbed per g of protein.

$$\text{WHC}\;(g/\text{g protein})=\frac{\text{Weight of pellet}\;(g)-\text{Weight of sample}\;(g)}{\text{Weight of sample}\;(g)\;\times \;C},$$

where *C* is protein concentration (%).

### 2.11 Protein solubility

The protein solubility of the samples was determined by the method of Park et al. (2016). 300 mg sample was dispersed in 30 ml of DDW and the pH of the mixture was adjusted to 2, 4, 6, 7, 8, 10, and 12, respectively, with 2 N HCl or 2 N NaOH. The pH adjusted mixture was stabilized at room temperature for 30 min and then centrifuged at 12,000 g for 20 min. The protein content in the supernatant was determined according to the Lowry's method (Lowry et al., 1951). Total protein content of the sample was measured by Lowry's method after solubilization of the sample in 2 N NaOH.

$$\text{Solubility}\left(\%\right)=\frac{\text{Protein content in supernatant}}{\text{Total protein content in sample}}\times 100$$

### 2.12 Foaming capacity and foam stability

The foaming capacity (FC) and foam stability (FS) of the 1% (w/v) sample dispersion were measured according to the method of Park et al. (2016). A quantity of 10 ml of 1% sample dispersion was transferred to a 25 ml volumetric cylinder and homogenized (POLYTRON^®^ PT 1200E, KINEMATICA AG, Luzern, Switzerland) at 12,500 rpm for 1 min at room temperature. The homogenized sample was allowed to stand for 0, 15, 30, and 60 min, respectively, and foaming capacity and foam stability were calculated using the following equations:

$$\text{Foaming capacity}\;\left(\%\right)=\frac{\text{VT}}{V_{o}}\times 100$$

$$\text{Foam stability}\;\left(\%\right)=\frac{\left(F_{t}/V_{t}\right)}{\left(\text{FT}/\text{VT}\right)}\times 100,$$

where VT is total volume after homogenizing; *V*_*0*_ is the original total volume before homogenizing; FT is foam volume after homogenizing; *F*_*t*_ and *V*_*t*_ are foam and total volume after leaving at room temperature for different times (*t* = 15, 30, and 60 min).

### 2.13 Emulsifying properties

The emulsifying activity index (EAI) and emulsion stability index (ESI) were measured according to the method of Park et al. (2016). Soybean oil (Ottogi Co., Ltd., Seoul, Korea) and 1% (w/v) dispersion sample at a ratio of 1:3 (v/v) were homogenized at a speed of 12,500 rpm for 1 min. Here, 50 μl of the emulsion was pipetted from the bottom of the volumetric cylinder at 0 and 10 min after homogenization and mixed with 5 ml of 0.1% SDS solution. The absorbance of the mixture was measured at 500 nm (UV‐2900, Hitachi, Kyoto, Japan). The absorbance measured immediately (*A*_0 min_) and 10 min (*A*_10 min_) after emulsion formations were used to calculate the emulsifying activity index (EAI) and the emulsion stability index (ESI) as follows:

$$\text{EAI}\;\left(m^{2}/\text{g protein}\right)=\frac{2\times 2.303\times A\times \text{DF}}{1\times \varphi \times C}\times 100,$$

where *A* = Absorbance 500 nm, DF = dilution factor (100), *l *= path length of cuvette (1 cm), φ = oil volume fraction (0.25) and *C* = protein concentration in aqueous phase (g/ml).

$$\text{ESI}\left(\text{min}\right)=\frac{A_{0}\times \Delta t}{\Delta A},$$

where Δ*A* = *A*_0 min_ – *A*_10 min_ and Δ*t* = 10 min. *A*_0 min_ and *A*_10 min_ are the absorbance measured immediately and after 10 min, respectively.

### 2.14 Statistical analysis

All experiments were performed at least three times and values were expressed as mean and standard deviation. The significant deference among the samples were analyzed by analysis of variance (ANOVA) and multiple ranges Duncan's test (*p *<* *.05) using the statistical software SPSS 12.0 K (SPSS Inc., Chicago, IL, USA).

## 3 RESULTS AND DISCUSSION

### 3.1 Proximate compositions

The results for the proximate composition and mineral contents of skipjack tuna roe (STR) and their roe protein concentrates are shown in Table 1. The yields of boil‐dried concentrate (BDC) and steam‐dried concentrate (SDC) per 100 g of STR were 22.4 g and 24.4 g, respectively. Protein yields of these concentrates were 16.4 g BDC and 18.5 g SDC, respectively, and the protein recovery for STR was 80.4% and 90.7%, respectively. The recovery rate of SDC is better than that of BDC as the steaming process loses relatively less solubles, including protein, than the boiling process. The decrease in protein recovery of BDC and SDC to STR was due to the soluble proteins and other organic compounds of roe that were liberated in the processed waters of the cook‐dried process (boiling or steaming). Intarasirisawat et al. (2011) reported that three species of tuna roes contained 72.2%–73.0% moisture, 18.2%–20.2% protein, 3.4%–5.7% lipid and 1.8%–2.1% ash. Lee et al. (2016) reported that the boil‐dried or steam‐dried roe concentrates of yellow fin tuna ranged from 4.8% to 5.8% in moisture content and from 76.0% to 77.3% in protein content, similar to the results of this experiment. As a positive control, egg white (EW) measured 3.4% for moisture and 81.2% for protein, and protein content was 5.2%–8.2% higher than skipjack roe concentrates. The fat content of STR was about 2%, which was about 35%–41% lower than that of the roe concentrates (3.1%–3.5%), reflecting the yield. These results indicate that the surface area of the concentrate powder is smaller than the surface area of STR, such that the extracted fat content of the STR is underestimated. The ash content of the STR was 1.1% and those of the concentrates reflecting the yield were 1.2% for BDC and 1.3% for SDC, respectively. Iwasaki and Harada (1985) reported the chemical composition of 18 species roes, and their protein content ranged from 11.5% to 30.2%. Narsing Rao et al. (2012) reported that Channa and Lates roes yielded 20.7% and 22.5% of protein concentrates containing 90.2% and 82.5% protein, respectively. Rodrigo, Ros, Periago, Lopez, and Ortuiio (1998) reported 39.1%–43.0% for protein content and 14.1%–14.8% for fat content in dried and salted roe of hake (*Merluccius merluccis*) and ling (*Molva molva*). In the above results and reports, the difference of moisture and protein content was due to processing conditions, with differences according to fish species (Mahmoud, Linder, Fanni, & Parmentier, 2008). The RPC powder prepared through the cooked and dried process (boil‐dried and steam‐dried) showed a high protein content (73%–76%) and its potential as a protein source was confirmed.

**Table 1:** Proximate composition and mineral contents of skipjack tuna roe and roe protein concentrates

Sample	STR	BDC	SDC	EW
Yield^a^ (g)	100.0	22.4	24.4	
Protein yield^b^(g)	20.4	16.4	18.5	
Moisture (%)	75.3 ± 0.2^a^	5.6 ± 0.1^c^	6.5 ± 0.0^b^	3.1 ± 0.6^d^
Protein (%)	20.4 ± 0.1^d^	73.0 ± 0.5^c^	76.0 ± 0.3^b^	81.2 ± 0.6^a^
Lipid (%)	1.9 ± 0.1^c^	15.6 ± 0.2^a^	12.7 ± 0.2^b^	ND
Ash (%)	1.1 ± 0.2^b^	5.4 ± 0.0^a^	5.2 ± 0.0^a^	ND
Minerals (mg/100g)				
K	355.0 ± 3.0^d^	763.2 ± 0.0^b^	707.5 ± 6.5^c^	795.0 ± 14.2^a^
S	322.2 ± 14.5^c^	853.2 ± 38.5^b^	816.6 ± 96.1^b^	1351.3 ± 10.2^a^
Na	191.0 ± 2.0^d^	278.6 ± 9.3^c^	371.6 ± 2.4^b^	1015.8 ± 8.8^a^
P	405.0 ± 3.0^a^	187.3 ± 36.1^b^	177.3 ± 1.6^b^	92.5 ± 0.4^c^
Mg	22.0 ± 0.0^b^	57.8 ± 6.3^a^	52.8 ± 0.6^a^	ND
Zn	8.0 ± 0.0^c^	45.5 ± 2.1^a^	37.2 ± 0.8^b^	ND
Ca	17.0 ± 0.0^d^	46.8 ± 0.2^b^	39.0 ± 0.4^c^	68.2 ± 0.6^a^
Fe	0.0 ± 0.0^d^	11.2 ± 0.5^a^	9.3 ± 0.1^b^	0.6 ± 0.0^c^

Data is given as mean values ± *SD* (*n* = 3). Means with different letters within the same row are significantly different at *p *<* *.05 by Duncan's multiple range test.

STR, skipjack tuna roe; BDC, boil‐dried concentrate; SDC, steam‐dried concentrate; EW, egg withe, respectively; ND, not determined.

a Yield is weight (g) of roe each sample obtained from 100 g of raw STR.

b Protein yield (g) = yield × protein (%).

### 3.2 Minerals

Mineral composition of RPCs was analyzed and the nutritional characteristics of the minerals as food compounds were examined (Table 1). The total mineral content of STR (1,129.2 mg/100 g) was almost equal to the ash content (1.1%), while those of BDC (2,243.6 mg/100 g) and SDC (2,210.8 mg/100 g) were significantly less than the ash content of BDC (5.4%) and SDC (5.2%). This is due to the fact that these minerals migrated from roes to process waters during the boiling and steaming process of the cook‐dried process. Phosphorus content was the most prominent mineral in STR (405.0 mg/100 g sample), followed by potassium (355.0 mg/100 g), sulfur (322.2 mg/100 g) and sodium (191.0 mg/100 g), respectively. Magnesium, calcium, and zinc content of STR as minor minerals were 22.0, 17.0 and 8.0 mg/100 g of STR, respectively. Heu et al. (2006) reported that major minerals in skipjack and yellow fin tuna roe were phosphorus (386.1 and 371.5 mg/100 g, respectively) followed by potassium and calcium. Lee et al. (2016) reported that the major minerals of yellow fin tuna roe are potassium (456 mg/100 g), phosphorus (437 mg/100 g) and sodium (167 mg/100 g), respectively. Major mineral contents of BDC and SDC were highest in sulfur (853.2 and 816.6 mg/100 g, respectively), followed by potassium, sodium, and phosphorus, respectively. In the mineral analysis of this experiment, the mineral content of BDC, with the exception of sodium, was significantly higher than that of SDC (*p* < .05). The highest sulfur content (1,351.3 mg/100 g) was found in EW as a positive control and was significantly higher than that of BDC and SDC (*p *<* *.05). Bekhit, Morton, Dawson, Zhao, and Lee (2009) reported that salmon roe had a sulfur content of 1,647–2,443 mg/kg (wet basis). From the results and reports, it is suggested that fish roes and egg white contain a large amount of sulfur‐contained compounds, which could be decomposed during storage, causing odors. EW had a lower phosphorus content (92.5 mg/100 g) than RPC (177.3–187.3 mg/100 g), but higher calcium content (68.2 mg/100 g). Thus, the ratio of calcium to phosphorus in the EW is ideal, while the phosphorus content of the STR and RPC is quite high. The phosphorus content has been generally associated with the phospholipid and the presence of phosphoprotein (Mahmoud et al., 2008). The content of magnesium (52.8–57.8 mg/100 g), zinc (37.2–45.5 mg/100 g) and iron in RPCs was higher than those of STR. In particular, iron content, which was not detected in trace amounts in STR, was found to be 9.3–11.2 mg/100 g in RPCs. These mineral analysis results showed similar trends to the results of yellowfin tuna roe and roe concentrates of our previous study (Lee et al., 2016). Variations in minerals of seafood products are closely related to seasonal trends, biological differences, catch areas, processing methods, food sources and habitats (salinity, temperature and pollutants) (Alasalvar, Taylor, Zubcov, Shahidi, & Alexis, 2002).

### 3.3 Total amino acids

STR and RPCs, which contained 77.3%–82.6% protein in dry base, still contained a significant amount of fish protein that could be used as a protein source. To evaluate protein quality, the total amino acid content (g/100 g of protein, %) of STR and RPCs was analyzed and compared with that of EW as a positive control (Table 2). The major nonessential amino acids (NEAAs) of STR were Glu (13.2%), Asp (9.0%), Ala (6.8%) and Ser (6.0%), respectively. Leu (8.3%), Lys (8.4%), Arg (6.6%) and Val (6.2%) were the major essential amino acids (EAAs) in STR. The ratio of EAA to NEAA in STR was found to be almost equal to 1.00. The major NEAAs of RPCs were Glu (12.7–12.8 g/100 g of protein), Asp (8.8%–9.1%) and Ala (6.7%–7.0%), respectively. Leu (8.5%–8.6%) was the predominant EAA followed by Lys (8.1%–8.4%) and Val (6.2%–6.5%). The ratios of EAA/NEAA in RPCs were 1.03 for BDC and 1.02 for SDC, respectively. From these results, the ratio of EAA/NEAA of STR and PRCs was lower than that of EW at 1.10, but similar to that of yellowfin tuna roe reported by Lee et al. (2016). Lysine is often the first limiting amino acid in cereal foods, and the lysine (8.1%–8.4%) content of RPCs was similar to that of EW (8.2%), suggesting that the protein quality of STR and its RPCs are excellent. Intarasirisawat et al. (2011) reported that leucine (8.3%–8.6%) and lysine (8.2%–8.3%) were the predominant EAAs in defatted tuna roes. Also, the leucine (8.5%–8.6%) and lysine (8.5%) content of yellowfin tuna roe and their roe concentrates (Lee et al., 2016) were similar to those of STR and RPCs. The content of hydrophobic amino acids (HAA) in STR and RPCs was 43.8%–45.2%, and there was no significant difference with that of EW (46.3%). Amino acid hydrophobicity plays an important positive role in determining emulsification characteristics for food functionality (Chalamaiah, Balaswamy, Narsing Rao, Prabhakara Rao, & Jyothirmayi, 2013). Thus, RPCs will be able to use dietary protein supplements for poorly balanced dietary proteins.

**Table 2:** Total amino acid content of skipjack tuna roe (STR) and roe protein concentrates

Amino acid^a^	STR	BDC	SDC	EW^c^
Protein content (%) (based on dry basis)	82.6	77.3	82.4	84.0
Thr	5.1^a^	5.0^b^	5.0^b^	4.7^c^
Val^b^	6.2^c^	6.5^b^	6.2^c^	8.2^a^
Met^b^	2.8^b^	2.8^b^	3.0^a^	2.0^c^
ILe^b^	5.1^d^	5.4^b^	5.2^c^	6.2^a^
Leu^b^	8.3^d^	8.5^b^	8.6^b^	9.2^a^
Phe^b^	4.1^c^	4.4^b^	4.5^b^	6.4^a^
His	3.5^a^	3.3^ab^	3.2^b^	2.7^c^
Lys	8.4^a^	8.4^a^	8.1^b^	8.2^b^
Arg	6.6^ab^	6.5^b^	6.7^a^	4.8^c^
EAA (%)	50.1	50.8	50.5	52.4
Asp	9.0^b^	9.1^b^	8.8^c^	11.8^a^
Ser	6.0^a^	5.7^b^	5.8^b^	5.7^b^
Glu	13.2^b^	12.7^c^	12.8^c^	14.9^a^
Pro^b^	5.8^b^	6.0^a^	6.0^a^	3.7^c^
Gly^b^	4.7^a^	4.6^ab^	4.5^b^	4.0^c^
Ala^b^	6.8^b^	7.0^a^	6.7^bc^	6.6^c^
Cys	1.1^a^	1.0^ab^	1.0^ab^	0.7^b^
Tyr	3.4^b^	3.1^c^	4.0^a^	0.2^d^
NEAA (%)	50.0	49.2	49.6	47.6
Total (%)	100.1	100.0	100.1	100.0
EAA/NEAA	1.00	1.03	1.02	1.10
HAA(%)	43.8	45.2	44.7	46.3

Data are means ± standard deviation of duplicate determination. Values with different letters within the same row are significantly different at *p* < .05 by Duncan's multiple range test.

STR, skipjack tuna roe; BDC, boil‐dried concentrate; SDC, steam‐dried concentrate; EW, egg withe; EAA, essential amino acids; NEAA, nonessential amino acids; HAA, hydrophobic amino acids.

a Amino acid (g/100 g protein) expressed a ratio of a kind of amino acid amount vs. total amino acid.

b Hydrophobic amino acid.

c Quoted from in our previous study (Lee et al., 2016).

### 3.4 SDS‐PAGE

**Figure 2:**
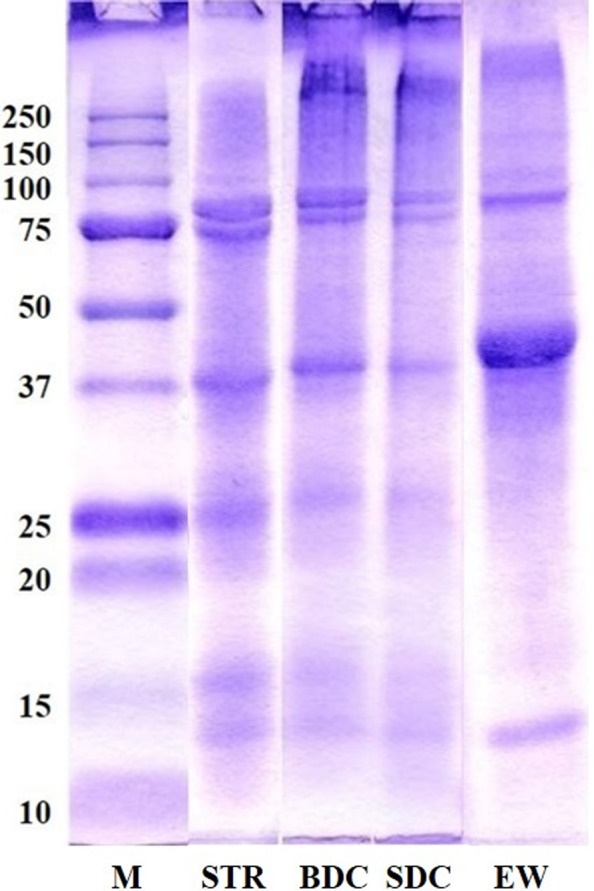
SDS‐PAGE patterns of protein concentrates prepared from skipjack tuna roe. M, protein maker; STR, skipjack tuna roe; BDC, boil‐dried concentrate; SDC, steam‐dried concentrate; EW, egg white

The SDS‐PAGE patterns of RPCs prepared from skipjack tuna roe are shown in Figure 2. STR and RPCs exhibited two clear bands in the range of 75–100 K. Protein bands of EW were observed in the range of 75–100 K, 37–50 K and 15 K, respectively, and clearer actin band was found in EW compared to STR. Protein bands around 97 K were estimated to be vitelline like proteins found in three species of tuna roes (Intarasirisawat et al., 2011) and egg yolk (Losso, Bogumil, & Nakai, 1993). The vitelline‐like protein bands of STR were more apparent than EW. The SDS‐PAGE patterns observed in BDC and SDC were similar to those of STR. However, protein bands above 250 K, which were not observed in STR, were clearly observed in BDC and SDC. These results may be due to protein coagulation or aggregation by heat treatments, such as boiling (BDC) and steaming (SDC), in the cook‐dried process. Protein bands ranging from 50 to 37 K, 37 to 20 K and 15 K were also observed, and these bands were estimated to be actin, troponin‐T and myosin light chain (MLC), respectively. Furthermore, proteins with 32.5 and 29 K were found in different tuna roes (Intarasirisawat et al., 2011). Those proteins were estimated to be ovomucoid (Al‐Holy & Rasco, 2006) or phosvitin (Losso et al., 1993). In the case of BDC and SDC, the protein bands in range of 15–25 K tended to decrease as compared to the STR bands. This is because some protein degradation and soluble low‐molecular proteins, such as sarcoplasmic proteins in skipjack tuna roe, were liberated into the process waters during the cook‐dried process. In the soluble fraction of sturgeon caviar, the protein of about 27 K is likely to be the ovomucoid as the 27–29 K glycoprotein (Al‐Holy & Rasco, 2006).

### 3.5 Buffer capacity

**Figure 3:**
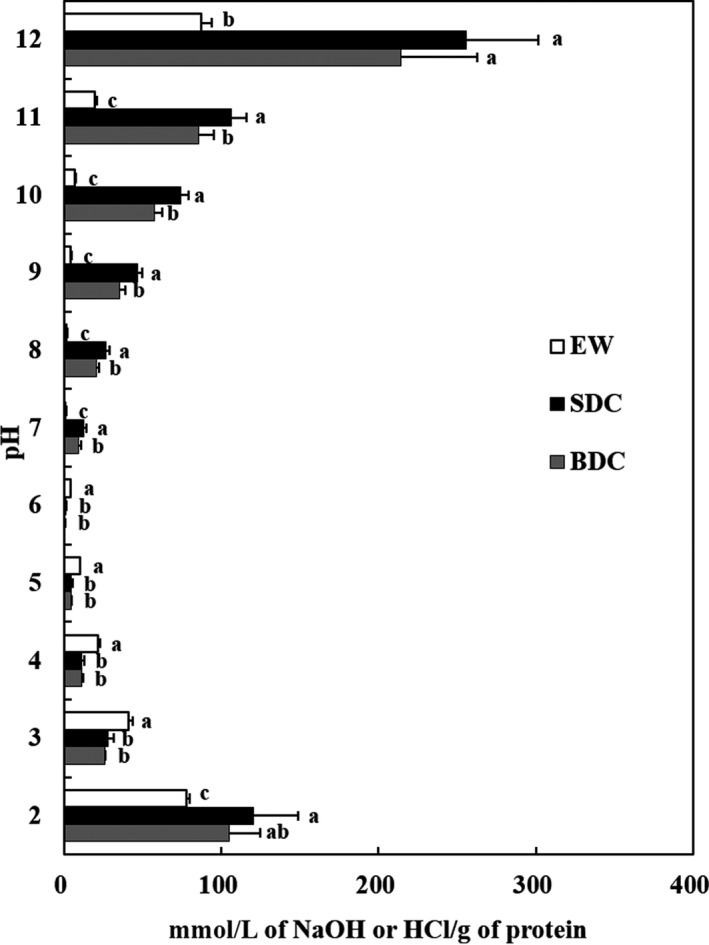
Buffer capacity of protein concentrates prepared from skipjack tuna roe. BDC, boil‐dried concentrate; SDC, steam‐dried concentrate; EW, egg white. Data are means ± standard deviation of triplicate determinations. Values with different letter within the samples are significantly different at *p *<* *.05 by Duncan's multiple range test

Buffer capacities of dispersed RPCs of BDC and SDC prepared with skipjack tuna roe are shown in Figure 3. The initial pH of the dispersed RPCs (1%, w/v) in DDW was 6.0 for BDC and 5.8 for SDC, respectively, and EW as a positive control was pH 7.4 (data not shown). At acidic pH in the pH range of 2–6, an average of 26.1 mmol/L and 29.9 mmol/L of HCl was required per g protein of BDC and SDC, respectively, to change the pH by 1 unit under these experimental conditions. In addition, in order to achieve a pH change of 1 unit in the pH range of 6–12, an average of 35.6 mmol/L and 42.6 mmol/L sodium hydroxide solution per 1 g of BDC and SDC was required, respectively. The buffer capacity of EW required an average of 19.5 mmol/L HCl and 15.2 mmol/L sodium hydroxide in the pH range of 2–6 and pH 6–12, respectively. BDC and SDC showed a higher buffer capacity than EW (*p *<* *.05) at pH‐shift range. In the cook‐dried process, the SDC showed a better buffer capacity than the BDC. This is because the leakage of the proteinous material into the process waters, which may affect the buffer capacity, was increased during the cooking process. Chalamaiah et al. (2013) reported that the initial pH of the dispersion of protein concentrates prepared from dehydrated or defatted mrigal (*Cirrhinus mrigala*) egg were noted as pH 5.5 and 5.8, respectively, and the buffer capacity of dehydrated protein concentrate was higher than that of defatted protein concentrate in both the acid and alkaline range, requiring an average of 0.65 mmol HCl and 1.22 mmol NaOH/1 g for one pH unit change. Narsing Rao (2014) reported that buffer capacities of protein concentrates (CRPC and ERPC) recovered from eggs of *Cyprinus carpio* and *Epinephelus tauvina* were higher in ERPC than in CRPC at acidic and alkaline pH ranges. Fish RPCs have been observed to have higher buffer capacity in alkaline than in acid, which means that a higher amount of alkali is needed when adjusting the pH for industrial use of protein concentrates (Park et al., 2016). It also means that it is stable against changes due to pH adjustment during processing. In the above results and literature, skipjack tuna roe concentrates showed higher buffering abilities and thus were considered to be highly applicable to various protein‐reinforced food materials.

### 3.6 Water holding capacity

**Figure 4:**
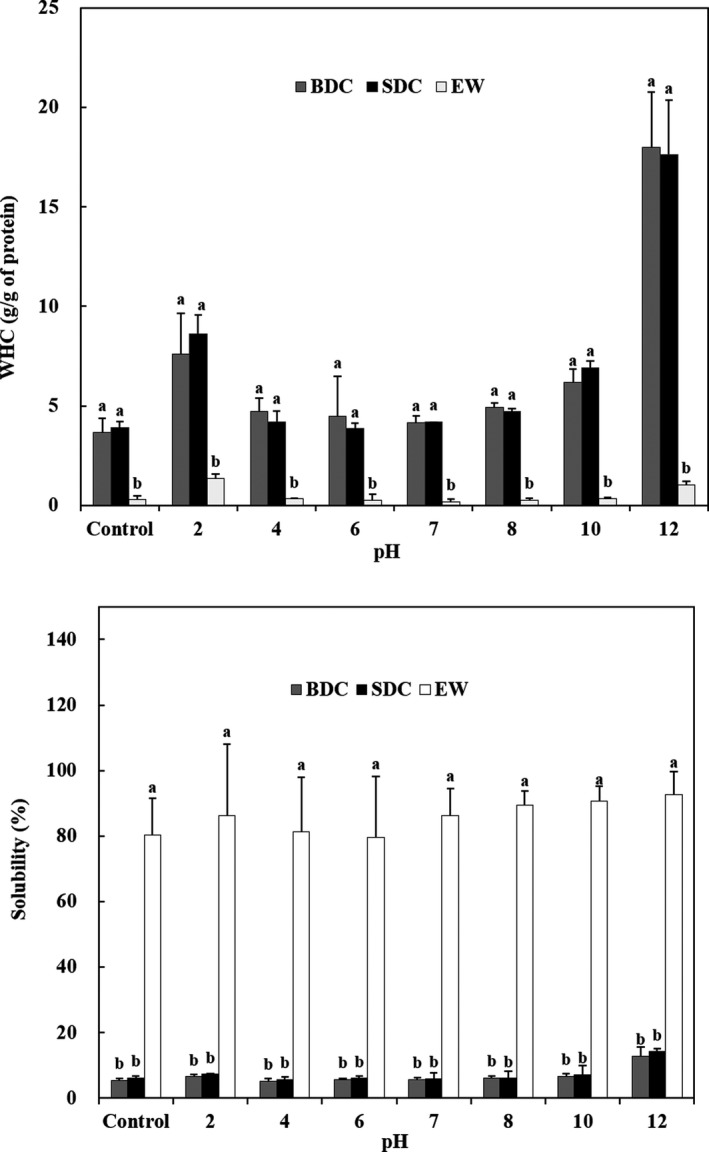
Water holding capacity (up) and protein solubility (down) of skipjack tuna roe protein concentrates (RPCs) without (control) and with pH‐shift. BDC, boil‐dried concentrate; SDC, steam‐dried concentrate; EW, egg white. Values represent the mean ± *SD* of *n* = 3. Data with different letters within the samples are significantly different at *p *<* *.05 by Duncan's multiple range test

Mohamed, Xia, Issoufou, and Qixing (2012) reported that proteins are important in food systems because of their effect on the taste, flavor, and texture of the food in the interactions of water and oil. However, WHC belongs to protein and food functionality, and is related to hydration. Thus, the WHC (g/g protein) of RPCs and EW with a pH‐shift in the pH range of 2–12 was measured and compared to controls without the pH‐shift, as shown in Figure 4 (up). The WHCs of BDC, SDC, and EW as controls without a pH‐shift were 3.7, 3.9, and 0.3 g/g protein, respectively. The WHC of BDC and SDC was not significantly different from each other, but EW value as a positive control was significantly lower than those of RPCs (*p *<* *.05). The pH‐shift treatment significantly improved the WHC of RPCs at all pHs except for pH 6 over controls without pH‐shifts. According to Li, Zhu, Zhou, and Peng (2011), when the protein is partially unfolded, it is likely that a flexible network is formed in which interactions between subunits are possible and water is captured. At pH 6, however, protein precipitation and aggregation increased, leading to a significant decrease in WHC (*p *<* *.05). Chalamaiah et al. (2013) reported that the water holding capacity (WHC) of defatted mrigal egg protein concentrate was high than that of Labeo rohita egg protein concentrate. Park et al. (2016) reported that the water holding capacity of yellow fin tuna roe concentrates was 4.1–4.7 g/g, which was superior to that of Labeo rohita (Balaswamy, Jyothirmayi, & Rao, 2007) and mrigal egg protein concentrates (Chalamaiah et al., 2013). The high WHC of protein concentrate may be due to the removal of fat components during the process (defatting, boiling and steaming), thereby increasing the polar groups such as hydrophilic COOH and NH_2_. These polar groups play an important role in water–protein interaction, and the exposure of polar groups to the surface of protein molecules affects the water holding capacity (Tan, Ngoh, & Gan, 2014).

### 3.7 Protein solubility

Good solubility is important in many protein‐based formulations because protein solubility is an important functional property affecting rheological, hydrodynamic, and surface activity (Yuan, Ren, Zhao, Luo, & Gu, 2012). The solubility of RPCs and EW after pH‐shift treatment at pH 2–12 is shown in Figure 4 (down) and compared with controls not subjected to pH‐shift treatment. There were no significant differences between the protein solubilities of BDC (5.5%) and SDC (6.0%) as controls without a pH‐shift, however, they were significantly lower than that of EW as a positive control (80.3%; *p *<* *.05). The solubility of pH shifted proteins is important for applications as a functional feature associated with protein and food processing systems, especially at pH <4 or >7 (Kinsella, 1976). BDC and SDC were unfolded and dissociated due to limited protein solubilization by acid and alkali, resulting in more hydrophobic residues being exposed and low solubility at all pH‐shift ranges (Balaswamy et al., 2007; Yuan et al., 2012). The solubilities of BDC (12.9%) and SDC (14.2%) were significantly lower than those of EW at pH 12 (*p *<* *.05). Park et al. (2016) showed that the solubility of yellowfin tuna roe concentrates at pH 12 was 8.6%–9.5%, which was somewhat lower than the results of this experiment. This is because boiling and steaming in the cook‐dried process caused exposure of hydrophobic moieties and thermal denaturation, which reduced protein solubility (Sikorski & Naczk, 1981). The high solubility of fish protein and their hydrolysates is an important feature in many food applications and positively affects other functional properties, such as foaming ability and emulsification.

### 3.8 Foaming capacity and foam stability

The foaming capacity (FC) and foam stability (FS) in food functionalities give the substance a unique character, such as refreshment, food softening, and dispersion of aromatic constituents. The FC and FS of 1% (w/v) RPCs are shown in Table 3. FCs of BDC and SDC dispersions before centrifugation were 111.7 and 107.9%, respectively, and that of EW was 128.2% (data not show). EW was significantly higher than BDC and SDC (*p *<* *.05). After centrifugation, the supernatant (126.6%) of the EW dispersion showed a higher FC than those of BDC (104.9%) and SDC (104.8%) (*p *<* *.05). Kudre and Benjakul (2013) reported that protein‐rich foam increases density and stability by increasing the interfacial layer thickness. In general, the foaming ability of proteins is related to their ability to form layers at the air–water interface. Proteins are rapidly adsorbed to the newly formed air/liquid interfacial layer during foaming, undergoing unfold and molecular rearrangement at the interface, resulting in an improved foam ability (Damodaran, 1997). After centrifugation, the supernatant of EW dispersion was observed to maintain high foam stability after whipping at 15 min (93.4%), 30 min (88.5%) and 60 min (84.9%), respectively. However, the FSs of BDC and SDC were not observed, regardless of centrifugation, and these foam layers disappeared immediately after whipping. This may be due to the coagulation or aggregation of the protein resulting from the heat treatment, which shows a lower solubility (Figure 4 down). The FCs of BDC (104.0%–118.2%) and SDC (104.0%–119.0%) were significantly lower than those (125.1%–172.3%) of EW at a pH‐shift range of 2–12 (*p* < .05; Table 3), and their FSs were not observed at all in the pH‐shift range of 2–10. At pH 12, however, RPCs showed a foam stability of 45%–72% up to 60 min. The FSs of EW showed high foam stability (71.9%–94.1%) up to 60 min in the entire pH‐shift range. RPCs showed the lowest foaming capacities at pH 4 and EW at pH 7, relating to the protein solubility (Figure 4 down). These pH values were estimated to be near the isoelectric point. These results suggest that the FC and FS of RPCs may be affected by protein solubility. It has been found that nonheat treatment processes, such as the freeze‐dried process (EW), are effective for high FC and FS compared to heat treatment processes (SDC and BDC). This result is similar to the foaming characteristics of yellowfin tuna roe concentrates (109%) and pH‐shifted foaming characteristics (Park et al., 2016).

**Table 3:** Foaming capacity (FC, %) and foam stability (FS, min) of skipjack tuna roe concentrates with pH‐shift

Sample	BDC	SDC	EW
Control			
FC (%)	104.9 ± 3.2^bB^	104.8 ± 1.4^bB^	126.6 ± 13.1^bA^
15min	–	–	93.4 ± 4.9
30min	–	–	88.5 ± 5.8
60min	–	–	84.9 ± 6.8
pH 2			
FC (%)	109.8 ± 3.9^bB^	106.0 ± 1.6^bB^	136.5 ± 5.0^bA^
60min	–	–	72.9 ± 4.1
pH 4			
FC (%)	104.0 ± 0.0^bB^	104.0 ± 0.0^bB^	131.0 ± 6.35^bA^
60min	–	–	76.4 ± 9.7
pH 6			
FC (%)	106.0 ± 3.2^bB^	106.5 ± 3.0^bB^	131.6 ± 16.9^bA^
60min	–	–	91.4 ± 5.3
pH 7			
FC (%)	106.2 ± 2.7^bA^	105.7 ± 2.2^bB^	125.1 ± 17.0^bA^
60min	–	–	91.7 ± 4.5
pH 8			
FC (%)	106.9 ± 2.1^bB^	105.2 ± 1.5^bB^	129.4 ± 17.0^bA^
60min	–	–	94.1 ± 8.9
pH 10			
FC (%)	107.6 ± 4.0^bB^	105.7 ± 2.1^bB^	133.4 ± 18.9^bA^
60min	–	–	91.2 ± 4.1
pH 12			
FC (%)	118.2 ± 9.5^aB^	119.0 ± 3.8^aB^	172.3 ± 28.1^aA^
15min	74.2 ± 18.1	71.5 ± 12.6	80.8 ± 2.3
30min	59.7 ± 14.4	64.6 ± 7.4	76.8 ± 2.4
60min	45.6 ± 12.1	47.9 ± 6.7	71.9 ± 3.2

Values represent the mean ± *SD* of *n* = 3. Means with different small letters within the same column and capital letters within same row are significantly different at *p *<* *.05 by Duncan's multiple range test.

BDC, boil‐dried concentrate; SDC, steam‐dried concentrate; EW, egg white.

### 3.9 Emulsifying properties

Emulsification is defined as the ability of a protein to adsorb oil to form an emulsion at the oil‐water interface, and emulsion stability is defined as the ability to stabilize the emulsion without forming adhesion and aggregation for the period of time (Can Karaca, Low, & Nickerson, 2011). The oil in water emulsifying activity index (EAI) and emulsion stability index (ESI) were performed to assess the ability to act as emulsifiers in a variety of foods, such as soups, sauces, confectionery breads, and dairy products. The EAI (m^2^/g protein) and ESI (min) of RPCs (1% water dispersion) are shown in Table 4. Before centrifugation, the EAI (m^2^/g protein) of the 1% dispersion of RPCs and EW as controls were 3.1 for BDC, 3.7 for SDC and 15.2 for EW, respectively (data not shown). The EAI (2.9 for BDC, 2.9 for SDC and 14.7 m^2^/g protein for EW) of these supernatants after centrifugation, tended to slightly decrease, however, did not show any significant differences (*p *>* *.05). The EAI (m^2^/g protein) of RPCs and EW were observed 2.3 for BDC, 11.1 for SDC and 18.0 for EW at pH 2, respectively, with significant differences (*p *<* *.05). BDC and SDC at pH 12 exhibited an EAI of 19.3 and 19.2 m^2^/g protein, respectively. BDC (1.4) showed the lowest emulsifying activity at pH 4, and SDC (1.9) at pH 6. In addition, it was shown that the nondenatured concentrate (EW) is superior to the cook‐dried concentrate (BDC and SDC) with an EAI. From the above results, it was confirmed that emulsifying activity is closely related to protein solubility by showing high emulsifying ability at extreme pH values (pH 2 and 12), where protein solubility is maximum (Mutilangi, Panyam, & Kilara, 1996). Thus, RPCs with high protein solubility could be rapidly diffused and adsorbed at the interface.

**Table 4:** Emulsifying activity index (EAI) and emulsion stability index (ESI) of roe protein concentrates with pH‐shift

Sample	BDC	SDC	EW
EAI (m^2^/g protein)			
Control	2.90 ± 0.81^b^	1.89 ± 0.26^b^	14.69 ± 0.67^a^
pH 2	2.32 ± 1.98^c^	11.05 ± 0.88^b^	18.03 ± 2.57^a^
pH 4	1.36 ± 0.73^c^	4.53 ± 0.76^b^	16.20 ± 1.23^a^
pH 6	3.89 ± 2.04^b^	1.86 ± 0.29^c^	16.89 ± 1.36^a^
pH 7	4.79 ± 1.83^b^	2.81 ± 0.61^b^	16.33 ± 1.62^a^
pH 8	5.15 ± 1.19^b^	3.21 ± 1.20^b^	16.57 ± 1.89^a^
pH 10	6.06 ± 1.65^b^	4.79 ± 0.13^b^	14.25 ± 1.88^a^
pH 12	19.31 ± 4.57^b^	19.22 ± 2.81^b^	26.16 ± 1.98^a^
ESI (min)			
Control	19.9 ± 7.8	29.5 ± 6.95	19.7 ± 3.30
pH 2	28.1 ± 6.0	14.4 ± 1.35	23.3 ± 3.89
pH 4	19.1 ± 0.7	16.8 ± 0.85	26.3 ± 6.79
pH 6	15.0 ± 1.5	34.8 ± 3.82	25.1 ± 6.91
pH 7	23.5 ± 4.7	40.7 ± 10.08	23.4 ± 6.66
pH 8	26.7 ± 5.7	28.5 ± 2.48	20.2 ± 2.98
pH 10	64.9 ± 8.8	68.1 ± 3.80	23.6 ± 5.96
pH 12	20.1 ± 6.0	18.1 ± 1.29	43.6 ± 8.06

BDC, boil‐dried concentrate; SDC, steam‐dried concentrate; EW, egg white.

Values represent the mean ± *SD* of *n* = 3. Means with different letters within the same column are significantly different at *p *<* *.05 by Duncan's multiple range test.

Roe protein concentrates with an EAI above 10 m^2^/g protein at various pHs showed an emulsion stability of 20.1 min for BDC (pH 12) and 14.4 and 18.1 min for SDC (pH 2 and 12, respectively). EW (23.3–43.6 min) showed higher emulsion stability than RPCs. Mutilangi et al. (1996) reported that a higher molecular weight or higher hydrophobic peptides content contributes to the stability of the emulsion. The mechanism of the emulsion formation is that the peptide is adsorbed on the surface of the newly formed oil droplet during the homogenization process, thereby forming a protective film to prevent adhesion of the oil droplets (Dickinson & Lorient, 1994).

Roe protein concentrates produced oil in water emulsions due to the action of hydrophilic and hydrophobic groups associated with surface active substances and their charge (Gbogouri, Linder, Fanni, & Parmentier, 2004). From these results, cook‐dried concentrates (BDC and SDC) prepared from skipjack tuna roe, which showed poor overall food functionalities, can improve food functionality by severe acid/alkali treatment. However, this can be a problem for food safety. Therefore, it is considered that the improvement of the food functionality, considering safety, can be attained by improving the solubility through preparing the enzyme hydrolysate. Enzymatic hydrolysis by proteases substantially improved the solubility of roe protein when compared to the solubility profile of un‐hydrolyzed roe proteins.

## 4 CONCLUSION

Roe protein concentrates prepared from skipjack tuna roe by the cook‐dried process improved the keeping quality in terms of moisture content (<7%) and yield (22.4%–24.4%). Because these RPCs are mainly composed of proteins (over 73%), they are considered to be highly suitable as a protein resource material. In addition, their buffering and water holding capacity were superior to egg white (EW), and was recognized as a valuable ingredient in protein‐fortified foods products. RPCs in the food and seafood processing industries will be available as egg white alternative protein sources and will be available as ingredients of fish sausage and surimi‐based products in particular. However, RPCs were found to be poorer in solubility, foaming and emulsifying properties than EW, which was caused by protein denaturation due to the cook‐dried process. Therefore, this study suggests that to improve the food functionality of RPCs, it is necessary to improve the solubility of RPCs such as enzymatic hydrolysis.

## CONFLICT OF INTEREST

The authors declare no conflict of interest.

## DECLARATION

This study has nothing to do with human and animal testing.
